# Fusion to Flaviviral Leader Peptide Targets HIV-1 Reverse Transcriptase for Secretion and Reduces Its Enzymatic Activity and Ability to Induce Oxidative Stress but Has No Major Effects on Its Immunogenic Performance in DNA-Immunized Mice

**DOI:** 10.1155/2017/7407136

**Published:** 2017-06-22

**Authors:** Anastasia Latanova, Stefan Petkov, Yulia Kuzmenko, Athina Kilpeläinen, Alexander Ivanov, Olga Smirnova, Olga Krotova, Sergey Korolev, Jorma Hinkula, Vadim Karpov, Maria Isaguliants, Elizaveta Starodubova

**Affiliations:** ^1^Engelhardt Institute of Molecular Biology, Russian Academy of Sciences, Moscow, Russia; ^2^Gamaleja Research Center of Epidemiology and Microbiology, Moscow, Russia; ^3^Department of Microbiology, Tumor and Cell Biology, Karolinska Institutet, Stockholm, Sweden; ^4^Chemistry Department, Belozersky Research Institute of Physico-Chemical Biology of Lomonosov Moscow State University, Moscow, Russia; ^5^Linköping University, Linköping, Sweden; ^6^Riga Stradins University, Riga, Latvia; ^7^M.P. Chumakov Institute of Poliomyelitis and Viral Encephalities, Russian Academy of Sciences, Moscow, Russia

## Abstract

Reverse transcriptase (RT) is a key enzyme in viral replication and susceptibility to ART and a crucial target of immunotherapy against drug-resistant HIV-1. RT induces oxidative stress which undermines the attempts to make it immunogenic. We hypothesized that artificial secretion may reduce the stress and make RT more immunogenic. Inactivated multidrug-resistant RT (RT1.14opt-in) was N-terminally fused to the signal providing secretion of NS1 protein of TBEV (Ld) generating optimized inactivated Ld-carrying enzyme RT1.14oil. Promotion of secretion prohibited proteasomal degradation increasing the half-life and content of RT1.14oil in cells and cell culture medium, drastically reduced the residual polymerase activity, and downmodulated oxidative stress. BALB/c mice were DNA-immunized with RT1.14opt-in or parental RT1.14oil by intradermal injections with electroporation. Fluorospot and ELISA tests revealed that RT1.14opt-in and RT1.14oil induced IFN-*γ*/IL-2, RT1.14opt-in induced granzyme B, and RT1.14oil induced perforin production. Perforin secretion correlated with coproduction of IFN-*γ* and IL-2 (*R* = 0,97). Both DNA immunogens induced strong anti-RT antibody response. Ld peptide was not immunogenic. Thus, Ld-driven secretion inferred little change to RT performance in DNA immunization. Positive outcome was the abrogation of polymerase activity increasing safety of RT-based DNA vaccines. Identification of the molecular determinants of low cellular immunogenicity of RT requires further studies.

## 1. Introduction

Starting from the first DNA immunization in 1991, multiple gene-based HIV vaccines have undergone preclinical and clinical trials [1–3]. Several of them that initially aimed to induce strong T cell responses failed to do so indicating a necessity to optimize both genes and their combinations. Several preclinical and clinical studies employed HIV *pol* gene, full-length or in fragments [4–6]. Plasmids encoding some of the *pol* gene products, as protease and integrase, were shown to be immunogenic in both preclinical and clinical trials [7–10]. At the same time, numerous trials showed an impaired immunogenicity of HIV-1 reverse transcriptase (RT) [11–13]. A recent study by Garrod et al. compared the performance in C57BL/6 mice of DNA vaccines encoding single HIV antigens in combination with HIV gag- and pol-based DNA immunogens. The efficacy of vaccination was tested by challenge with a chimeric EcoHIV virus that can infect mice [14]. At 60 days, there was significantly lower frequency of induced antigen-specific CD8(^+^) T cells in the spleens of pCMVgag-pol-vaccinated mice compared with mice immunized with single pCMVgag. Furthermore, while short-term viral control of EcoHIV was similar for gag- and gag-pol DNA-vaccinated mice, only gag DNA-vaccinated ones were able to control EcoHIV two months postvaccination, indicating that inclusion of the HIV *pol* gene may reduce the durable control over viral replication [14].

HIV enzymes encoded by *pol* gene, including RT, are crucial if aiming at immunotherapeutic vaccination which would prevent drug resistance in HIV infection [15]. Potent immunogenic performance of all three HIV enzymes is a prerequisite of the efficacy of such immunotherapy. We and others performed series of studies aimed to improve the immunogenicity of RT, a key enzyme determining HIV-1 resistance to antiretroviral therapy, but with a limited success [12, 13, 16–18]. Lately, we found that cells expressing HIV-1 RT produce reactive oxygen species (ROS) and express high levels of phase II detoxifying enzymes that interfere with the immune response against this enzyme [19, 20]. Oxidative stress is induced by a wide panel of RT variants, drug-resistant, expressed from viral and expression-optimized genes, enzymatically active and inactive [19] indicating that the ability to induce oxidative stress and oxidative stress response is a property of a domain (domains) within the protein rather than the consequence of its enzymatic activity. We hypothesized that cellular immunogenicity of HIV RT in DNA immunization may be increased by decreasing the levels of this stress-inducing protein in the expressing cells.

We tested if this is the case by artificially promoting RT export. For this, we provided a multidrug-resistant variant of HIV-1 RT (RT1.14) [16], complemented for safety sake, with mutations inhibiting polymerase and RNase H activity, with a leader signal peptide (Ld) of the nonstructural protein 1 of tick-borne encephalitis virus (NS1 of TBEV). NS1 is synthesized as a monomer and dimerizes after the posttranslational modification; it is also expressed on the cell surface and is secreted as a hexamer [21–23]. Ld peptide is responsible for the presentation of NS1 on the cellular surface and further secretion [24–26]. We characterized the properties of Ld-RT1.14 chimera such as the half-life, route of degradation, efficacy of secretion, capacity to induce oxidative stress, and oxidative stress response and, finally, studied its performance in DNA immunization in a mouse model. Retargeting of RT to ER with subsequent secretion resulted in an increase in the RT expression levels due to protein stabilization and also, interestingly, in the nearly complete inhibition of the residual polymerase activity retained in the inactivated RT. Secretion led to a mild reduction of oxidative stress, but no significant enhancement of the cellular immune response in the experimental DNA immunization. Immune response to RT remained tinted towards the production of RT-specific antibodies, typical to M2 polarization of macrophages and Th2 polarization of T-cells in the settings of oxidative stress [27–29].

## 2. Materials and Methods

### 2.1. Plasmids

Expression-optimized gene encoding reverse transcriptase derived from the patient infected with multidrug-resistant HIV-1 clade B isolate (RT1.14, [18]) with mutations D185N, D186N, and E478Q abrogating polymerase and RNase H activities was cloned into pVax1 vector (Invitrogen, USA) generating pVaxRT1.14opt-in which was described by us earlier [19]. Sequence encoding RT1.14opt-in was used as a backbone to design a chimeric gene encoding RT1.14 with the N-terminal insertion of the leader sequence of NS1 protein of TBE. The latter was cloned from plasmid pLdNS1 carrying the gene of NS1 protein of Western European subtype of TBE [24]. The cloned fragment encoded 25 amino acids of a leader sequence of NS1 and the N-terminal methionine for an effective initiation of translation. This fragment including 78 nucleotide b.p. was amplified with KAPA HiFi Hot Start DNA polymerase (Kapa Biosystems, USA) and Ld-NheI-F (5′-ATA-CGC-AAG-CTA-GCA-ATA-TGA-GAA-ACC-CTA-CAA-TG-3′) and Ld-BamHI-R (5′-TCT-AAC-AGG-ATC-CCG-CCC-CCA-CTC-CAA-GGG-3′) primers (Syntol, Russia), containing restriction sites *NheI* and *BamHI*, respectively. Resulting PCR product was cloned at the 5′-terminus of RT1.14 gene using *NheI* and *BamHI* restriction sites. Fusion of NS1 Ld- and RТ1.14opt-in-encoding fragments formed one open-reading frame encoding optimized inactivated leader-fused RT1.14, dubbed RT1.14oil, within the plasmid pVaxRT1.14oil. Nucleotide sequence of the cloned fragment was confirmed by sequencing. For immunization, plasmids were purified using Plasmid Endofree kits (Qiagen, Germany) as described by the manufacturer.

### 2.2. Expression of Homo- and Heterodimers of Reverse Transcriptase in *E. coli*

For expression in *E. coli* genes of active and inactivated RT, HIV-1 HXB2 and RT1.14 were cloned into a two-cistron vector pET-2c. Proteins were expressed in *E. coli* and purified by ion exchange chromatography as homodimers using the protocol described earlier [30]. Wild-type p66/p51 heterodimeric HIV-1 RT was expressed in M-15 [pREP4] *E.coli strain* transformed with the plasmid p6HRT [31] and purified as previously described [32].

### 2.3. RT Polymerase Activity Assay

The RT assays using activated DNA were performed as follows: the standard reaction mixture (20 *μ*l) contained 0,75 *μ*g of activated DNA, 0,02–0,05 *μ*g RT, 3 *μ*M dATP, 30 *μ*M of dCTP, dGTP, and dTTP, 1 *μ*Ci [a-32P]dATP in a Tris-HCl buffer (50 mM, pH 7,5) containing also 10 mM MgCl_2_, and 0,2 M KCl. The reaction mixtures were incubated for 12 minutes at 37°C and applied onto Whatman 3MM filters with a 0,5 M EDTA solution to stop reaction. After drying on air, the filters were washed twice with 10% trichloracetic acid, then twice with 5% trichloracetic acid, and once with ethanol and dried on air. The radioactivity was measured by the Cherenkov method [33]. Radioactivity was determined using a Tri-Carb 2810 TR scintillation counter (Perkin Elmer, USA).

### 2.4. RNase H Activity Assay

RNase H activity assay of HIV-1 RT was tested by using 6,7 mmol of 18-ribo-Fl/18-deoxy duplex (18-mer oligoribonucleotide (18-ribo-Fl: 5′-r(GAUCUGAGCCUGGGAGCU)-fluorescein-3′) and 18-mer oligodeoxyribonucleotide (18-deoxy d5′-d(AGCTCCCAGGCTCAGAUC)-3′)). RNA/DNA duplex was added to reaction mixtures consisted of 15 *μ*L of 50 mM Tris/HCl (pH 8,0), containing 60 mM KCl, 10 mM MgCl_2_, and various concentrations of p66/p51 RT variant (5, 20, 100, and 400 nM) followed by a 15 min incubation at 37°C. The reaction was stopped by adding 80 *μ*l of 7 mM EDTA, 0,375 M sodium acetate, 10 mM Tris-HCl (pH 8,0), and 0,125 mg/ml glycogen. The mixture was extracted by phenol/chloroform, and RNA/DNA fragments were precipitated with ethanol. The reaction products were separated by electrophoresis in a 20% polyacrylamide/7 M urea gel (PAGE). Gel images were recorded using Typhoon FLA 9500TM Phosphorimager (Molecular Dynamics, Israel) and then quantified using Quantity One 4.6.6. (Bio-Rad, USA). The experiments were conducted three times.

### 2.5. Eukaryotic Cell Cultivation and Transfection

HeLa and HEK293T cells were cultivated at 37°C in 5% CO_2_ in DMEM medium (PanEco, Russia) supplemented with 10% of fetal bovine serum (FBS; HyClone, USA) and 100 *μ*g/ml of streptomycin/penicillin mixture. Transfection was performed with Lipofectamine LTX (Invitrogen, USA) in accordance with manufacturer's instructions. Cells were grown for 48 hours, cells were harvested, and cell culture fluids were collected and used for further analysis of RT expression, studies of oxidative stress, and RT polymerase activity.

### 2.6. Immunostaining of RT Expressing Eukaryotic Cells

HeLa cells were grown and transfected on the cover glass (20 × 20 mm) in the 6-well cell culture plates (Corning, Costar, USA). Two days posttransfection, cells were fixed with methanol-acetone (1 : 1). After fixation, cells were incubated in the staining buffer (PBS, 2% BSA, 0,2% Tween 20, and 10% glycerol) containing monoclonal murine anti-RT (1 : 10) or polyclonal rabbit anti-RT (1 : 100) and subsequently stained with FITC-conjugated anti-murine or anti-rabbit antibodies (1 : 50) (Dako, Denmark) as described previously [12]. Cell nuclei were stained with DAPI fluorochrome (Invitrogen, USA). For surface protein analysis, cells were fixed with 2% paraformaldehyde, then blocked with 5% BSA in PBS, and incubated with anti-RT antibodies diluted in the staining buffer without Tween 20. Slides were read on the confocal microscope Leica TCS5 (Leica, Germany).

### 2.7. Quantification of RT Expression in Eukaryotic Cells and Cell Culture Fluids by Western Blotting

RT1.14-expressing HeLa were lysed and analyzed by Western blotting using polyclonal rabbit anti-RT [34] or monoclonal murine anti-RT antibodies [35] as primary and anti-rabbit or anti-mouse HRP-conjugated antibodies as secondary (Jackson, USA) as was previously described [12]. Cell culture fluids were centrifuged at 5000 ×g and then concentrated approximately 10 times with Vivaspin 500 units (Sartorius Stedim, Germany) with a 30,000 MWCO membrane. Concentrated culture fluid was mixed with Laemmli buffer and then subjected to Western blotting same way as the cell lysates. Immune complexes on the membrane were detected with ECL (Amersham, USA) and X-ray film (FujiFilm, Japan). The data was processed in ImageJ software (http://rsb.info.nih.gov/ij). After RT-specific staining, blots were washed and restained first with monoclonal anti-*β*-actin murine antibodies (Sigma, USA) and then with anti-mouse HRP-conjugated antibodies (Dako, Denmark). To assess the level of RT expression per cell, the percent of cells expressing RT was estimated from the efficacy of transfection established in a control cotransfection with GFP plasmid (peGFP-N1, Novagen, Germany) used as a reporter. The number of cells for each sample was counted with hemocytometer and certain number of cells was taken for Western blotting analysis. The number of transfected cells was estimated from the percentage of transfection efficacy. The amount of RT protein in the lysed cells and culture fluids was calculated based on a standard curve built using the recombinant RT 1.14 protein and dispensed in serial dilutions in the concentration range from 1 to 20 ng per well; the latter samples were analyzed together with the lysates as described earlier [12]. RT content per cell was calculated by dividing these values by the number of transfected cells.

### 2.8. Quantification of RT Polymerase Activity in Cell Lysates and Cell Culture Fluids

Two days posttransfection of HeLa cells with pVaxRT1.14opt-in or pVaxRT1.14oil plasmids, cell culture fluids were collected and concentrated as described above. Cells were lysed in TNEV buffer containing 50 mM Tris HCl (pH 7,5), 1% Triton X-100, 2 mM EDTA, and 100 mM NaCl supplemented with protease inhibitor cocktail (Sigma, USA). RT activity in the cell lysates and cell culture fluids was assessed by Lenti RT activity kit (Cavidi, Sweden) following instructions of the manufacturer. The amount of protein determined by the polymerase activity assay was normalized to the total amount of RT protein assessed by Western blotting using the calibration curve built with the recombinant RT1.14 protein in a concentration range from 1 to 20 ng per well. The data obtained represented the relative polymerase activity of the samples.

### 2.9. Measurement of Reactive Oxygen Species

Measurement of reactive oxygen species was performed as described earlier [36]. In brief, 40 hours posttransfection, HEK293T cells were incubated for 30 min in cell culture medium containing 25 *μ*M 2′,7′ dichlorodihydrofluorescein diacetate (DCFH). Fluorescence intensities were measured using Plate CHAMELEON V reader (Hidex Ltd., Finland) with the excitation at 485 nm and emission at 535 nm.

### 2.10. Reverse Transcription and Quantitative PCR (RT-qPCR)

RNA was isolated from 5 × 10^5^ transfected HEK293T cells with PerfectPure RNA Cultured Cell kit (5 Prime, Germany, USA) and then transcribed with Reverse Transcription System (Promega, USA) with random hexamer primer according to manufacturer's protocol. RT-qPCR was performed using iQ5 Real-Time PCR Detection System (BioRad, USA) and primers and probes which were described earlier [36]. A standard reaction mixture (50 *μ*l) contained Taqman primer/probe combination, cDNA equivalent to 100 ng of total RNA, and qPCR-HS master mix. The thermal conditions for PCR reaction for all genes were 55°C for 5 min, 95°C for 10 min followed by 40 cycles at 95°C for 10 sec, and 57°C for 1 min (signal collection temperature). Relative quantitative analysis was performed by comparing threshold cycle number for target genes and a reference *β*-actin mRNA, amplified in separate tubes.

### 2.11. Degradation of RT in Eukaryotic Cells

The measurement of half-life of the protein was done using the cycloheximide chase assay based on the method described earlier [37]. For this, HeLa cells 48 hours posttransfection were treated with cycloheximide (Sigma-Aldrich, USA) at the final concentration 100 *μ*g/ml. After 0, 2, 4, and 6 hours of incubation, the cells were harvested, lysed, and analyzed by Western blotting. The half-life time for the protein was calculated with a standard formula: *Т*_1/2_ = −0,693 t/ln*N*/*N*_0_, where *N_0_* is the initial amount of protein and *N* the amount of protein at time *t*. To evaluate the role in RT degradation of the proteasome and lysosome inhibitors, HeLa cells were transfected with pVaxRT1.14opt-in or pVaxRT1.14oil and 24 hours posttransfection treated with the cellular protease inhibitors at the final concentrations indicated in the brackets: E64 (10 *μ*M), leupeptin (10 *μ*g/ml), aprotinin (10 *μ*g/ml), pepstatin A (7,5 *μ*M), MG132 (5 *μ*M), or epoxomicin (0,1 *μ*M) (all from Calbiochem, USA). After 18 hours of incubation with the inhibitors, cells were lysed and the residual amount of RT was estimated by Western blotting.

### 2.12. DNA Immunization of Mice

Eight-week-old BALB/c mice (8 weeks, Charles River Laboratories, Sandhofer, Germany) were housed under a light-dark (12 h/12 h) cycle with ad libitum access to water and food. Animals were anesthetized by a mixture of 4% isoflurane with oxygen and maintained in 2,3% isoflurane flow administered through facial masks during all intradermal injections and electroporation. All experimental procedures were approved by the local ethical committee. Groups consisting of 4–6 mice were injected with 10 *μ*g of pVaxRT1.14opt-in or pVaxRT1.14oil, or with empty pVax1 vector, mixed with equal amount of pVaxLuc encoding firefly luciferase administered with 29G insulin needles in 20 *μ*l of PBS. Each mouse got two intradermal DNA injections, to the left and to the right of the base of the tail. Immediately after, injection sites were subjected to electroporation using DermaVax DNA vaccine delivery system (Cellectis Glen Burnie, France) as described earlier [38]. In day 21 postimmunization, mice were bled and sacrificed and their spleens were collected.

### 2.13. Analysis of Cellular Immune Response

#### 2.13.1. INF-*γ* and IL-2 Fluorospot

The spleens of immunized mice were homogenized, and the splenocytes were isolated as described in [8]. The cells were incubated in RPMI medium supplemented with 2 mM L-glutamine, 100 *μ*g/ml of streptomycin/penicillin mixture (all from Sigma-Aldrich, USA), and 10% FBS (Gibco, Invitrogen, USA) (complete media), with the following antigens taken in concentration 10 *μ*g/ml: synthetic peptides corresponding to RT aa375-389 (ITTESIVIWGKTPKF), 465-476 (KVVPLTNTTNQK), 514-528 (ESELVNQIIEQLIKK), 528-543 (KEKVLAWVPAHKGIG), leader sequence of NS1 (RNPTMSMSFLLAGGLVLAMTLGVGA) (all peptides from GL Biochem Ltd., China) and RT 1.14 protein. Concanavalin A (ConA, 5 *μ*g/ml) was used as a positive control and cell culture medium as a negative control. After 20 hours of incubation, IFN-*γ* and IL-2 secretion by splenocytes was assessed by dual IFN-*γ*/IL-2 Fluorospot (Mabtech, Sweden) in accordance with the protocol provided by the manufacturers. The number of cells secreting cytokines was calculated with fluorimeter AID ELISpot (Autoimmun Diagnostika GmbH, Germany).

#### 2.13.2. Detection of Secreted Granzyme B and Perforin by Splenocytes

Isolated murine splenocytes were applied on 96-well V-plates in 200 *μ*l RPMI-10% FBS and stimulated in duplicates with the recombinant RT1.14 protein and RT peptides, same as used in Fluorospot test (10 *μ*g/ml). Cells were incubated for 3 days at 37°C and 5% CO_2_. After that, 100 *μ*l of cell culture fluid from each well was collected, duplicates were pooled, redivided into two 100 *μ*l aliquots, and subjected to analysis by sandwich ELISA for granzyme B (DuoSet Development kit; R&D Systems Europe Ltd.) and perforin (PRF1; Hölzel Diagnostika Handels GmbH, Germany). Kits were used as recommended by the manufacturers.

### 2.14. Detection of Anti-RT Specific Antibodies by Indirect ELISA

96-well microtiter plates (Nunc Maxisorp, Denmark) were coated with recombinant RT1.14 protein or NS1 Ld peptide diluted in PBS at 0,3 *μ*g/ml by overnight incubation at 4°C. Solution was discarded, and plates were washed with PBS containing 0,05% Tween 20. Individual mice sera diluted in HIV-scan buffer (2% normal goat serum, 0,5% BSA, 0,05% Tween 20, and 0,01% sodium merthiolate) in five (IgA sybtype) or three (IgG subtypes) fold-steps starting from 10 (IgA) or 200 (IgGs) were applied on the plates and incubated overnight at 4°C. The plates were washed as above, and HRP-conjugated goat anti-mouse IgG or IgA (Sigma, USA) diluted in HIV-scan buffer was applied and incubated for 1,5 hours at 37°C. After the incubation, plates were washed as above and color reaction was developed with 3,3′,5,5′- tetramethylbenzidine substrate solution (TMB; Medico-Diagnostic Laboratory, Russia). Reaction was stopped by addition of 50 *μ*l of 2,5 M of sulfuric acid, and optical density was recorded at a dual wavelength of 450 and 650 nm. The cutoff for specific RT antibody response was set as mean OD values showed by sera of vector-immunized mice at this time point +3 SD. For positive sera showing OD values exceeding the cutoff, endpoint dilution titers were established from the titration curves.

### 2.15. In Vivo Monitoring of Reporter Expression

Bioluminescence emission from the area of DNA-immunogen/Luc gene injection was analyzed days 1, 3, 9, 15, and 22 postimmunization. To estimate luciferase expression in vivo, mice were intraperitoneally injected with the solution of D-luciferin (Perkin Elmer, USA) in PBS in 15 mg/ml at a dose of 100 *μ*l for 10 g of body weight and left for 5 minutes. In vivo imaging of bioluminescence was performed with a highly sensitive CCD camera, mounted in a light-tight chamber (Spectrum CT, Perkin Elmer, USA). Anesthesia was induced by 4% isoflurane and maintained by 2,3% isoflurane throughout the imaging procedure. Camera exposure time was automatically determined by the system and varied between 1 and 60 s depending on the intensity of the bioluminescent signal. Regions of interest were localized around the injections sites and were quantified as the total luminescence flux in photons/s. CT/BLI data were processed using the Living Image® software version 4.1 (Perkin Elmer, USA) to generate values of total photon flux and mean photon flux (photons/sq cm) from the injected area.

### 2.16. Statistics

Statistical evaluations were done using STATISTICA AXA 10.0 (StatSoft Inc., OK, USA). Nonparametric statistics was chosen as appropriate for sample sizes <100 entries. Continuous but not normally distributed variables, such as the average radiance in photons/s/cm^2^/Sr, antibody titers, or the number of cytokine-producing SFCs, cytokine levels in pg/ml, were compared in groups by the nonparametric Kruskal-Wallis and pairwise by Mann–Whitney *U* test. Correlations were run using the Spearman rank order test. *p* values <0.05 were considered significant.

## 3. Results

### 3.1. Design and Expression of the Inactivated Multidrug Resistant RT Fused to the Leader Peptide of TBEV NS1 (RT1.14oil)

The secretable RT chimera was designed based on the plasmid pVaxRT1.14opt-in [19] which encodes an inactivated multidrug-resistant RT variant (RT1.14opt-in). Original RT1.14 amino acid sequence originated from HIV-1 clade B isolate from patient with multiple drug resistance [18] modified by mutations introducing two D/N substitutions in the polymerase YMDD motif, and one E/Q substitution in the RNase H DEDD motif, shown earlier to inhibit respective enzymatic activities [39–42]. Inactivation of the polymerase and RNase H moieties of RT was confirmed in in vitro assays done on the “classical” RT derived from HIV-1 HXB2 strain and its analogue inactivated by D185N, D186N, and E478Q mutations, represented, for adequate characteristics of the enzymatic activity, by p51/p66 heterodimers (Supplementary Figure S1 available online at https://doi.org/10.1155/2017/7407136). Introduction of D185N and D186N mutations completely abrogated the polymerase activity of the enzyme (Figure S1A). We have also assessed the RNase H activity of the active and inactivated RTs (Figure S1B, C). The wild-type RT demonstrated significant activity in a broad range of enzyme concentrations tested (Figure S1B, C). The efficacy of hydrolysis of the 18-mer RNA/DNA hybrid duplex strongly depended on the enzyme concentration (Figure S1B, C). On the contrary, RT inactivated with mutation E478Q in the RNase H moiety was practically inactive even at high enzyme concentrations; the efficiency of hydrolysis was reduced by over 90% compared to the wild-type enzyme. These results confirmed the adequacy of inactivation strategy used to generate the inactivated drug-resistant HIV RT.

**Figure 1:**
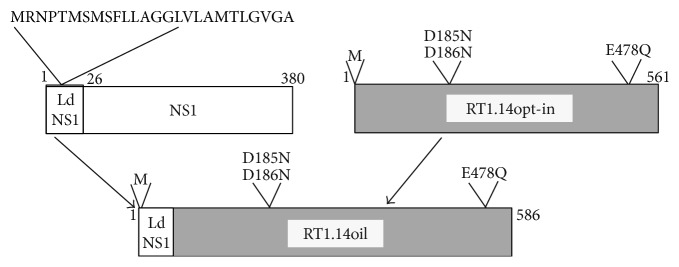
Schematic representation of chimeric drug-resistant HIV-1 reverse transcriptase carrying the N-terminal insertion of the N-terminal signal peptide of NS1 protein of TBEV (RT1.14oil). Rectangular boxes stand for polypeptide chains of NS1 with leader signal peptide (white) and RT1.14opt-in sequence (gray) with amino acid substitutions in polymerase (D185N, D186N) and RNase H signature motives (E478Q) leading to inactivation of respective enzymatic activities in the resultant RT1.14oil polyprotein. Amino acid positions in the polypeptide chain are designated with numbers.

To design the secreted form of inactivated RT1.14, we used a specialized signal of the heterologous viral protein, namely of the nonstructural protein 1 of tick-borne encephalitis virus (TBEV NS1) [24] (Figure 1). The leader signal of TBEV NS1 (Ld) consisting of the last 25 amino acid residues of TBEV envelope protein (glycoprotein) E is responsible for the transport to ER and secretion of the downstream NS1 protein [25, 26]. DNA sequence encoding LdNS1-RT1.14opt-in chimera was generated by PCR and cloned into pVax1 vector yielding a plasmid dubbed pVaxRT1.14oil (Figure 1).

**Figure 2:**
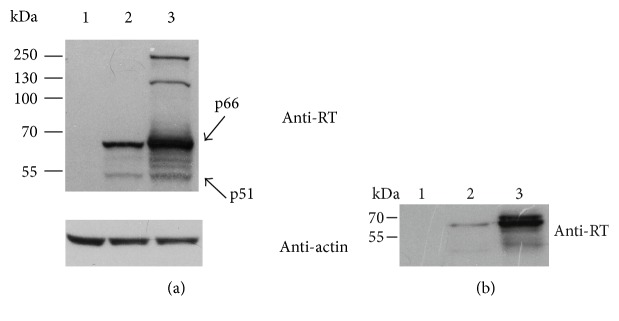
Expression of HIV-1 RT1.14 fused to the N-terminal signal peptide of NS1 protein of TBEV in HeLa cells resolved by SDS-PAGE. (a) Western blotting of the lysates of HeLa cells transfected with pVax1 (1), pVaxRT1.14opt-in (2), and pVaxRT1.14oil (3) plasmids; upper panel, membranes stained with anti-RT; lower panel, with anti-*β*-actin antibodies for signal normalization. (b) Western blotting of the culture fluids of cells transfected with pVax1 (1), pVaxRT1.14opt-in (2), and pVaxRT1.14oil (3). Blots were stained with the specific polyclonal anti-RT antibodies as described in the experimental section “Materials and Methods.” To control equal loading of the samples of cell lysates on the gel, membranes were for the second time stained with anti-*β*-actin antibodies. Positions of the molecular mass markers are given to the left.

We have then compared the expression profiles of the parental inactivated RT1.14 and of Ld-RT1.14 chimera. For this, we transfected HeLa cells with pVaxRT1.14opt-in and pVaxRT1.14oil plasmids; 48 hours posttransfection, cell lysates and cell culture fluids were collected and analyzed by Western blotting. Lysates of cells from both transfections contained proteins with molecular mass of 66 kDa coinciding with the expected molecular mass of RT1.14 [43] (Figure 2(a)). The amount of the 66 kDa product detected in the lysates of cells transfected with pVaxRT1.14oil was approximately 1,4 times higher than in the lysates of cells transfected with the parental RT1.14opt-in gene (Figure 2(a)). Both lysates contained also a protein with the molecular mass of 51 kDa corresponding the polymerase subunit of HIV-1 RT [44]. Besides, lysates of cells transfected with pVaxRT 1.14oil contained products of higher molecular mass (over 170 kDa) that were specifically stained with anti-RT antibodies; suggestively, the aggregates formed as a result of RT overexpression. RT1.14opt-in was found in the cellular fraction and, interestingly, in equal amounts, in the cell culture fluid from the expressing cells (Table 1). Ld-RT1.14 chimera encoded by pVaxRT1.14oil was preferentially (>70%) found in the cell culture fluid (Table 1).

### 3.2. Enzymatic Activity of RT in the Expressing Cells and in the Culture Fluids

Next, we analyzed the lysates of cells transfected with RT1.14opt-in and RT1.14oil genes for the polymerase activity of RT by an ELISA-based test (Cavidi, Sweden). We also collected, concentrated, and subjected to the HIV RT activity test samples of the cell culture fluids. RT protein content in these probes was estimated by Western blotting.

A residual RT polymerase activity was detected in all probes, but not in the probes from nontransfected HeLa cells. We evaluated the specific RT activity per 10^5^ of HeLa cells transfected with RT1.14opt-in and with RT1.14oil coding plasmids. Lysates of 10^5^ cells expressing RT1.14opt-in had RT activity corresponding to 9,8 pg of the active protein, that is, retained 0,00012% activity (9,8 pg of the active form in the total 8,3 *μ*g of the enzyme by Western blotting; Table 1). Lysates of 10^5^ cells expressing RT1.14oil retained 0,00005% of the activity (5,7 pg of the active form in the total 11,3 *μ*g of the enzyme; Table 1). At the same time, cell culture fluids of RT1.14opt-in expressing cells retained 0,00053% of the activity (74,4 pg out of 14 *μ*g), which is four times more than the specific activity in the cells (Table 1). The latter pointed that RT1.14opt-in is secreted in the active form even without the signal peptide. On contrary, cell culture fluids of RT1.14oil-expressing cells contained only 0,000014% of the active enzyme (4,9 pg per 34,6 *μ*g), that is, 3,5 times less than the lysates of RT1.14oil-expressing cells (Table 1). Thus, RT1.14oil was effectively secreted, but almost exclusively in the inactive form.

### 3.3. RT Localization in the Expressing Eukaryotic Cells

**Figure 3:**
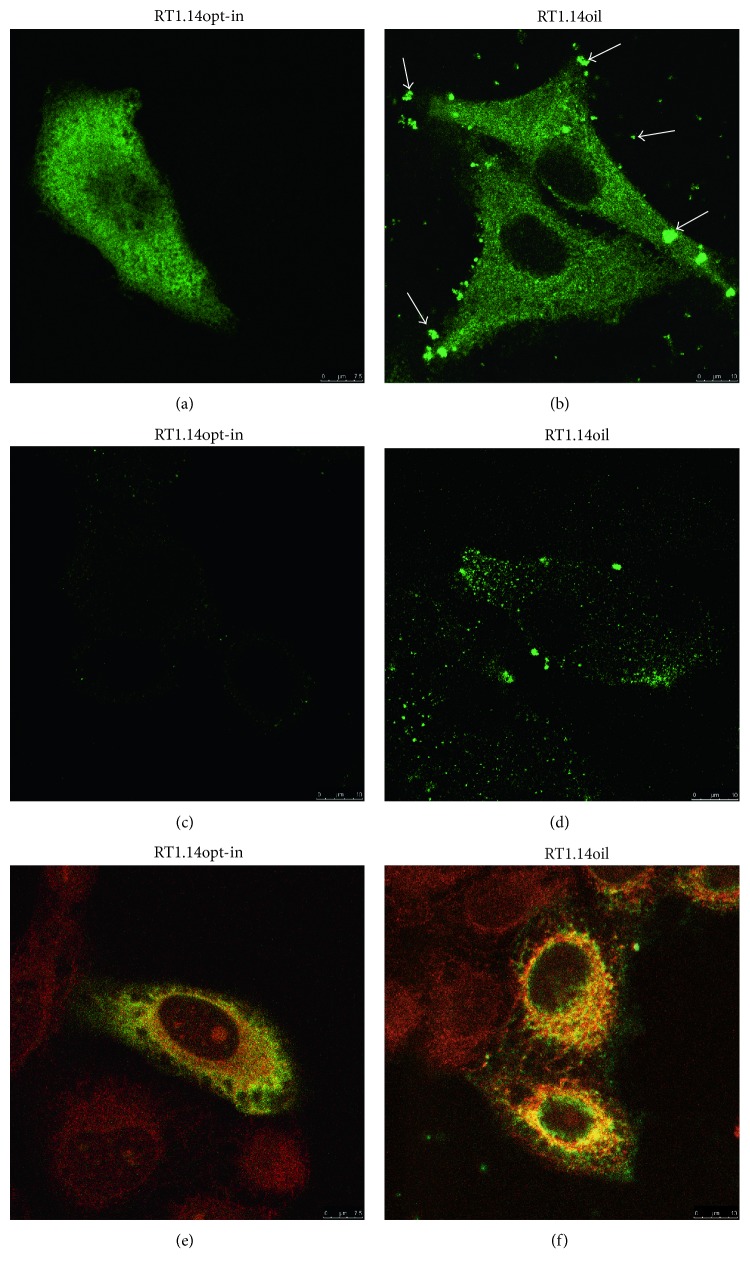
Cellular localization of RT without signal of secretion (RT1.14opt-in) and with a signal peptide (RT1.14oil) in HeLa cells. HeLa cells were transfected with the plasmids pVaxRT1.14opt-in (a, c, e) and pVaxRT1.14oil (b, d, f), grown on slides for 48 hours, fixed with methanol : acetone (1 : 1 *v*/*v*) (a, b, e, f) or 2% paraformaldehyde (c, d), and stained first with polyclonal anti-RT and with secondary FITC-conjugated anti-rabbit antibodies. For localization of RT in relation to ER, HeLa cells were treated with TRITC-conjugated antibodies to an ER marker calreticulin (e, f), and the overlay of FITC and TRITC signals is shown.

Intracellular localization of RT variants was investigated by the immunofluorescence microscopy. HeLa cells were transfected with pVaxRT1.14opt-in and pVaxRT1.14oil plasmids and subsequently stained with anti-RT and then FITC-conjugated secondary antibodies. RT1.14opt-in was distributed in cell cytoplasm homogeneously (Figure 3(a)), whereas the distribution of RT1.14oil was more granular (Figure 3(b)). Besides, cultures of the cells transfected with pVaxRT1.14oil plasmid were characterized by the presence of granules specifically stained with anti-RT antibodies that were localized inside and outside the cells and on the cell surface (Figure 3(b), indicated by arrows). To confirm the cell surface localization of the protein, transfected cells were fixed with 2% paraformaldehyde with subsequent staining in the absence of detergent. Surface of the cells transfected with the initial plasmid pVaxRT1.14opt-in was not specifically stained (Figure 3(c)), whereas the surface of pVaxRT1.14oil-transfected cells was specifically stained with anti-RT antibodies (Figure 3(d)). To localize RT in relation to ER, HeLa cells were treated with TRITC-conjugated antibodies to an ER marker calreticulin, and FITC and TRITC signals were overlaid (Figures 3(e) and 3(f)). In the case of RT1.14oil, RT-specific signal was partially colocalized with ER marker and partially with the secretory vesicles, whereas RT1.14opt-in had a more prominent colocalization with ER.

### 3.4. RT Protein Degradation

**Figure 4:**
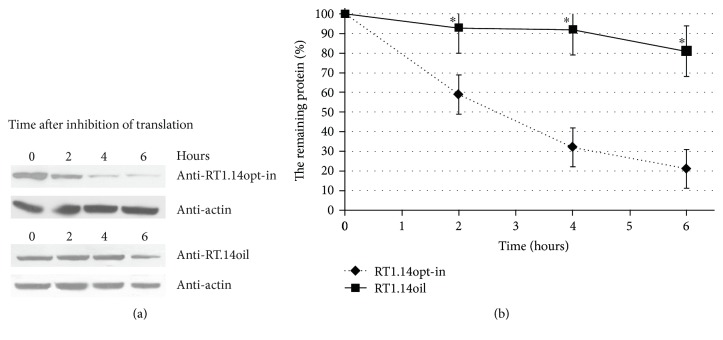
The kinetics of degradation of RT1.14opt-in and RT1.14oil in HeLa cells after blocking of translation with cycloheximide. (a) Western blotting of HeLa cells transfected with pVaxRT1.14opt-in and pVaxRT1.14oil plasmids 0, 2, 4, and 6 hours after cycloheximide treatment. The blots were stained with polyclonal anti-RT antibodies. To control equal loading of the samples on the gel, membranes were restained with anti-*β*-actin antibodies. (b) Diagram of protein degradation speed. 100% is according to initial amount of the protein. The graph is plotted in accordance with the results of 3 independent experiments, ±SD. ^∗^*p* < 0,05 (Mann–Whitney test).

Next, we estimated the speed of degradation of RT1.14oil in HeLa cells and investigated the contribution of cellular proteases into its proteolysis. To estimate the half-life of the protein, cells transiently expressing RT1.14oil, or RT1.14opt-in, were after two days treated with cycloheximide and lysed 0, 2, 4, and 6 hours after the on-start of the treatment. The amount of RT1.14opt-in and RT1.14oil in the probes was detected by Western blotting (Figure 4(a)). The half-life of RT1.14opt-in was estimated as 2 and of RT1.14oil as more than 8 hours (approximately 15 hours) which characterizes it as a long-living protein (Figure 4(b)).

**Figure 5:**
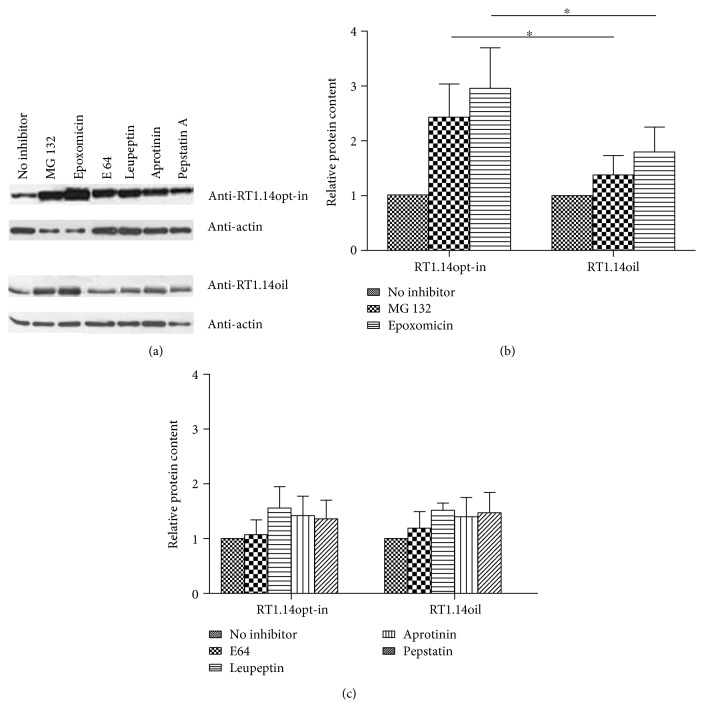
RT accumulation in HeLa cells treated with inhibitors of proteasomal and lysosomal proteolysis. Western blotting of HeLa cells transfected with pVaxRT1.14opt-in and pVaxRT1.14oil after 18-hour incubation with MG132 (5 *μ*М), epoxomicin (0,1 *μ*М), E64 (10 *μ*М), leupeptin (10 *μ*g/ml), aprotinin (10 *μ*g/ml), and pepstatin А (7,5 *μ*М) or without inhibitors. The blots were stained with monoclonal anti-RT antibodies. To control equal loading of the samples on the gel, the membranes were stripped and restained with anti-*β*-actin antibodies (a). Relative content of RТ1.14opt-in and RТ1.14oil in the samples treated with proteasomal inhibitors (b). Relative content of RТ1.14opt-in and RТ1.14oil in the samples treated with lysosomal inhibitors; no difference in response to lysosomal inhibitors between RT1.14opt-in- and RT1.14oil-expressing cells was detected (all *p* values > 0,05) (c). In (b) and (c), protein content in the untreated samples is taken for 1. Graphs in (b) and (c) represent the results of three independent experiments, +SD. ^∗^*p* < 0,05 (Mann–Whitney test).

For a detailed study of RT proteolysis, on the next day after the transfection, cells were treated with the inhibitors of proteasomal and lysosomal proteolysis. The role of proteasome in degradation was assessed using reversible (MG132) and irreversible (epoxomicin) proteasomal inhibitors. Lysosomal proteolysis was blocked with the inhibitors of cysteine (E64, leupeptin), serine (aprotinin, leupeptin), and acid proteases (pepstatin A). Cells were incubated with the inhibitors for 18 hours and lysed, and RT content was estimated by Western blotting in comparison with the content in the untreated samples (Figure 5(a)). Treatment with MG132 increased RT1.14opt-in content 2,5, and with epoxomicin, 3 times compared to untreated samples; lysosomal inhibitors had no effect. None of the inhibitors had any effect on the intracellular level of RT1.14oil (Figures 5(b) and 5(c)).

### 3.5. Induction of Oxidative Stress and Oxidative Stress Response in RT1.14-Expressing Cells

**Figure 6:**
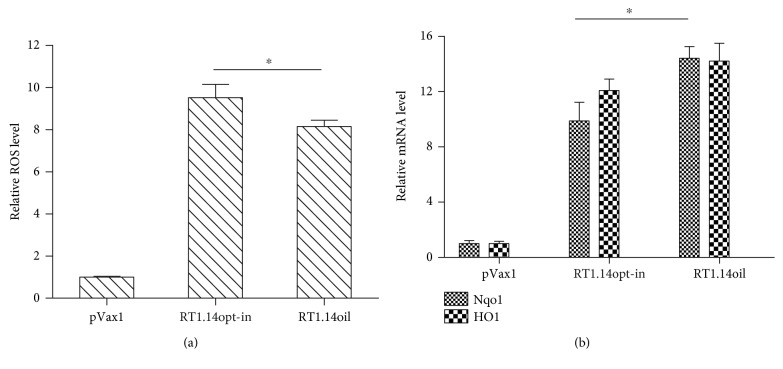
Induction of oxidative stress and oxidative stress response in cells expressing multidrug-resistant inactivated reverse transcriptase RT1.14opt-in and its derivative RT1.14oil carrying a signal peptide. Induction of the oxidative stress was detected as the production of ROS (a) and increase in the levels of mRNA of the phase II detoxification enzymes Nqo1 and HO1 (b). Levels of ROS were normalized to those in HEK293T cells transfected with the empty vector pVax1. Levels of mRNA for Nqo1 and HO-1 were normalized to levels of mRNA for actin and then represented as fold difference to the effect of empty vector pVax1. Data represent the results of two independent experiments, each done in triplicate, +SD. Results are compared using *F* test (Statistica AXA 10), ^∗^*p* < 0,05.

Next, we have evaluated the level of oxidative stress induced by RT1.14opt-in and RT1.14oil in HEK293T cells transiently transfected with their genes. Oxidative stress was measured by the formation of reactive oxygen species (ROS) visualized in the presence of fluorogenic 2, 7′-dichlorofluorescein. The presence of both RT variants in cells led to the formation of ROS (Figure 6(a)). Interestingly, secretion resulted in a weak but significant decrease in the levels of ROS production (15%; *p* = 0,013; *F* test; Figure 6(a)). Next, we have studied the induction of oxidative stress response, namely, the induction of expression of phase II detoxifying enzymes, heme oxygenase 1 (HO1), and NAD(P)H-oxygenase 1 (Nqo1). Level of transcription of their genes was evaluated by RT-PCR. Expression of RT variants led to an increase in the levels of expression of both detoxifying enzymes (Figure 6(b)). Cells expressing RT1.14oil demonstrated an increase in the expression of Nqo1 (*p* < 0,05; *F* test; Figure 6(b)) and a tendency to the increased induction of HO1 (*p* = 0,07; *F* test; Figure 6(b)).

### 3.6. RT1.14 Performance in DNA-Immunized Mice

**Figure 7:**
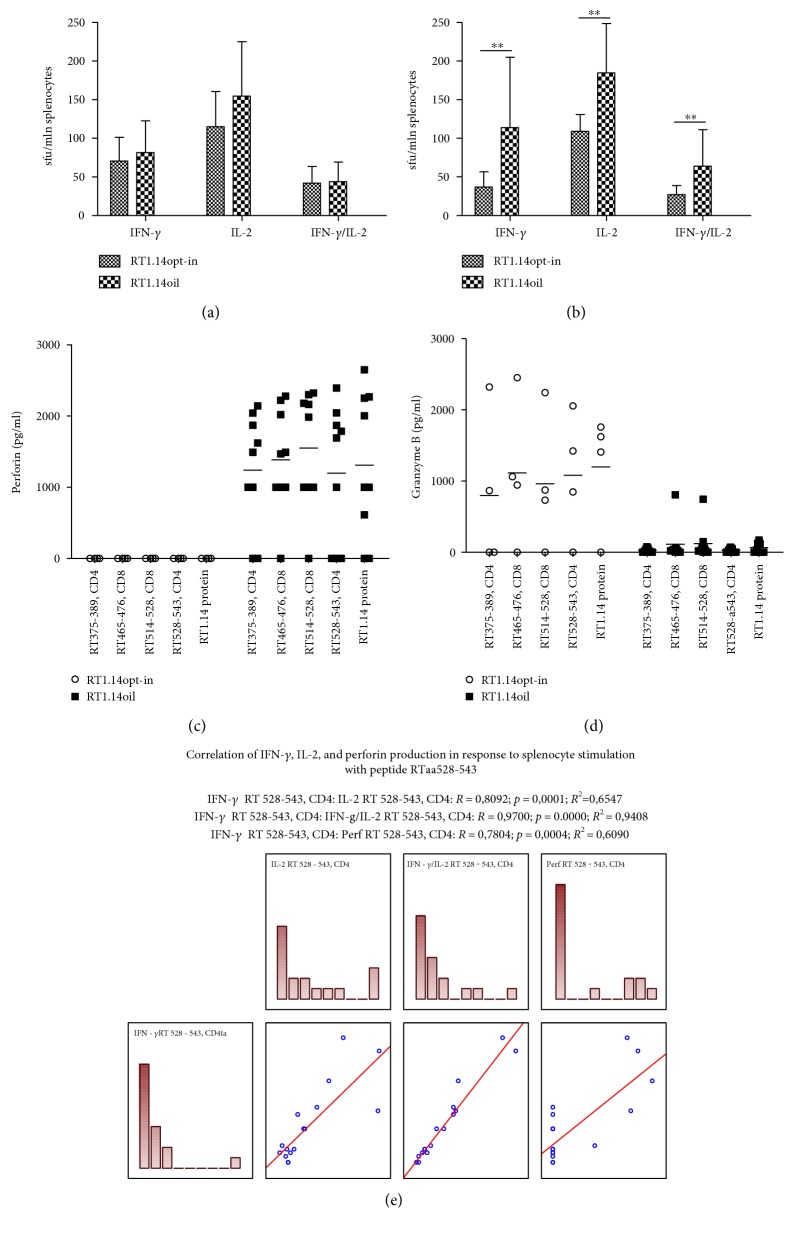
Cellular immune response of mice immunized with pVaxRT1.14opt-in and pVaxRT1.14oil. IFN-*γ*, IL-2, and dual (IFN-*γ*/IL-2) secretion by splenocytes in response to stimulation with RT1.14 protein (a) or RT1.14-derived peptide aa528-543 (b) measured by Fluorospot. Responses represent the average number of signal-forming units (sfu) per mln cells in two independent experiment runs, each done in duplicate, +SD, *n* = 4, ^∗∗^*p* < 0,1 (Mann–Whitney test). Secretion of perforin (c) and granzyme B (d) by splenocytes of mice immunized with pVaxRT1.14opt-in and pVaxRT1.14oil in response to stimulation with RT1.14 protein and RT1.14-derived peptides. Splenocytes were stimulated with the recombinant RT 1.14 protein and RT peptides for 3 days. After that, cell culture fluids were collected and subjected to the analysis for granzyme B and perforin by sandwich ELISA. Data represent an average value of two repeated measurements for each mouse, in pg/ml. Correlation of the parameters of cellular immune response (e).

BALB/c mice were injected with pVaxRT1.14opt-in, pVaxRT1.14oil, or pVax1 plasmids intradermally with subsequent electroporation. On day 22, mice were sacrificed; blood and spleens were collected. Splenocytes were isolated and tested for the ability to proliferate after in vitro stimulation with RT-derived antigens. Immune response was evaluated as the production of IFN-*γ* and IL-2 by Fluorospot and perforin and granzyme B by sandwich ELISA. RT1.14opt-in- and RT1.14oil-immunized mice demonstrated similar levels of cellular response against the recombinant RT1.14 protein (Figure 7(a)). Immunization with DNA encoding RT1.14opt-in and RT1.14oil induced a weak IFN-*γ* and IL-2 response against a CD4^+^ T cell epitope of RT localized at aa 528-543 [45], which tended to be stronger in mice DNA immunized with RT1.14oil (Figure 7(b), *p* < 0,1). Peptides representing other known epitopes of RT induced no specific IFN-*γ* or IL-2 production (data not shown). Interestingly, splenocytes of mice DNA immunized with RT1.14oil responded to stimulation with these peptides by a strong production of perforin (Figure 7(c)). Levels of perforin secretion significantly correlated to the levels of RT-specific production IFN-*γ* and IL-2 (Figure 7(e); *R* values >0,8; *p* < 0,0005). On contrary, mice DNA immunized with RT1.14opt-in responded to stimulation with RT-derived peptides by a high-level production of granzyme B which was not correlated to either IFN-*γ* or IL-2 levels (Figure 7(d), data not shown). IFN-*γ*/IL-2 responses have also been tested in splenocytes stimulated with a peptide encoding a leader sequence of NS1 (NS1 leader peptide). No specific cytokine secretion was detected (data not shown).

**Figure 8:**
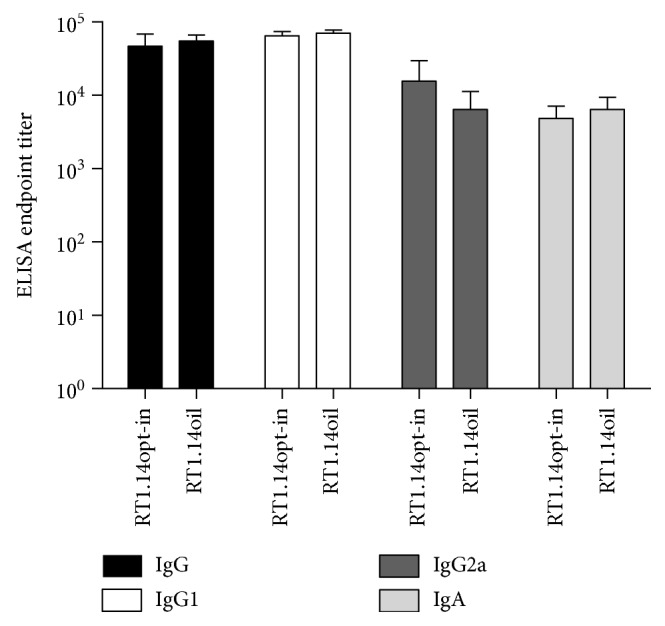
RT-specific IgGs and IgA induced in sera of mice immunized with pVaxRT1.14opt-in and pVaxRT1.14oil plasmids. Endpoint titers of RT-specific total IgG, IgG subtypes (IgG1, IgG2a), and IgA antibodies detected in the sera of BALB/c mice immunized with the genes encoding RT1.14opt-in and RT1.14oil plasmids. Data represent a mean + SD for the endpoint titer of antibodies against recombinant RT1.14 protein for four mice per group in two independent immunization runs. Cutoffs were set against the control mice immunized with empty vector pVax1 (see “Materials and Methods”). Groups demonstrate no significant difference in either IgG, or IgG1, or IgG2a, or IgA levels (*p* > 0,05).

Murine sera collected at the endpoint of the experiment were analyzed for anti-RT antibodies by indirect ELISA. No significant difference was detected in antibody titers reached in both groups of RT-immunized mice. Titers of IgG and IgG1 exceeded 50,000 in both groups, IgG2a reached 15,000 in RT1.14opt-in and 6000 in RT1.14oil-immunized mice and IgA 5000 in RT1.14opt-in and 6000 in RT1.14oil-immunized mice, respectively (Figure 8). Ratio of IgG2a/IgG1 was equal to 0,15 in both groups of mice indicating a Th2 type of immune response [46]. TBEV NS1 Ld peptide induced no specific antibodies (data not shown).

### 3.7. Effector Immune Response to RT and Its In Vivo Evaluation

**Figure 9:**
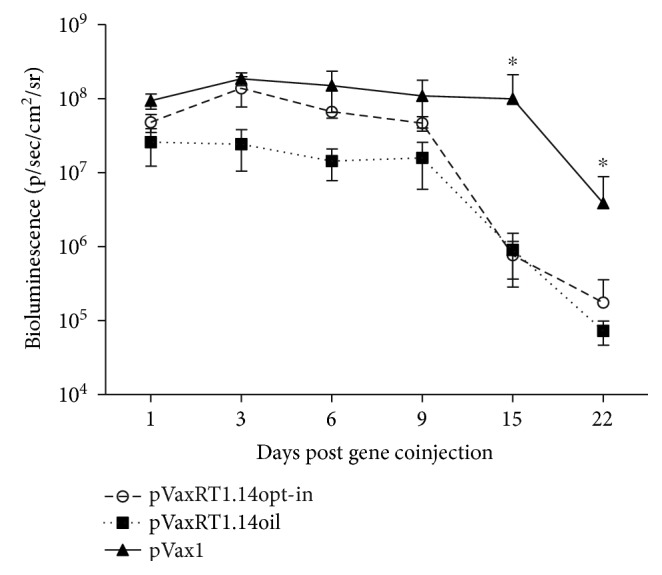
Kinetics of in vivo bioluminescence from the sites of coadministration of RT genes and luciferase reporter genes. In vivo monitoring of luciferase activity on days 1, 3, 6, 9, 15, 22 after the administration of plasmids encoding RT1.14opt-in and RT1.14oil, or empty vector pVax1, each mixed with pVaxLuc encoding firefly luciferase (1 : 1 *w*/*w*). One curve represents bioluminescent emission from eight mice with two immunization sites in each, assessed in two independent immunization runs. ^∗^*p* < 0,05 (Mann–Whitney test).

We have earlier shown that diminishment of bioluminescence induced by the expression of reporter gene coinjected with DNA immunogen correlates with the induction of polyfunctional cellular responses against the immunogen [8, 47] specifically with IFN-*γ*/Il-2 responses of CD4 T cells [8]. To monitor this effect, mice were coinjected with RT gene variants and a reporter gene encoding firefly luciferase (Luc) and assessed for the levels of bioluminescence on days 1, 3, 6, 9, 15, and 22 postimmunization. We have observed a significant diminishment of in vivo luminescence after day 9 in all immunized mice, independently of the enzyme form (Figure 9()).

## 4. Discussion

Multiple efforts made by us, and by others, to enhance the immunogenicity of HIV-1 RT had limited success [12–14, 16–18]. Recently, we discovered that both the wild-type and drug-resistant RT variants induce oxidative stress in the expressing cells [19]. Oxidative stress has a dual impact on the development of immune response, and low to moderate levels of stress may be stimulating, whereas excessive stress affects both the innate and adaptive immune response [48–51]. High levels of oxidative stress detected in the RT-expressing cells during DNA immunization may suppress the development of specific immune response. We hypothesized that this effect can be neutralized or minimized by an early export of RT from the expressing cells. To test this, we supplemented RT with the leader signal peptide of NS1 protein of TBEV, responsible for cotranslational translocation of the carrier proteins into ER with subsequent secretion [25, 26]. As the backbone for this chimera, we chose the most immunogenic RT variant of the ones we have tested so far, RT of HIV-1 clade B strain with multiple mutations of drug resistance (RT1.14) [18] encoded by the expression optimized gene. RT1.14 was devoid of the enzymatic activities by two D/N mutations in YMDD, and one E/Q mutation in DEDD motives was shown to inhibit the polymerase and RNase H activities, respectively [39–42]. We have proven that these mutations successfully abrogate both activities of the model RT of HIV-1 HXB2 strain. A chimera of the inactivated multidrug-resistant RT with the leader peptide Ld of NS1 dubbed “Optimized Inactivated with Leader sequence” RT1.14, or RT1.14oil, was thus created.

Fusion of the leader peptide sequence changed localization of the chimera. The distribution of RT1.14oil was more granular in comparison to RT1.14opt-in. Also, in the case of RT1.14oil, RT-specific signal was partially colocalized with the ER marker and in part with the secretory vesicles, unlike the parental RT1.14opt-in which had a more prominent ER colocalization. RT1.14oil was detected in the cells and as granules on the cell surface and in the intercellular space. As expected, RT1.14oil was detected also in the cell culture fluids with three times more protein there than in the cells. Altogether, this demonstrated the success of our strategy of RT export. Leader peptide of TBEV NS1 can function as an efficient signal for targeting the heterologous proteins into the secretory pathway. Detection of RT1.14oil both in the cells (cell lysates) and in the cell culture fluids was an expected finding. We have also made an unexpected finding of RT1.14opt-in not only inside the cells (in cell lysates) but also in the cell culture fluids. One of the explanations could be the leakage from apoptotic cells dying due to oxidative stress. However, we have not observed an abnormal/excessive cell death of the RT-expressing cells neither in this nor in the earlier studies [19]. The actual RT export/secretion mechanism requires further elucidation.

Relocalization of RT1.14oil drastically changed its processing. Firstly, its half-life increased from 2 to >8 hours. While RT1.14opt-in was mainly degraded by proteasome, RT1.14oil was insensitive to most of the cellular proteases tested. Thus, retargeting of RT1.14oil into the secretory pathway led to its “escape” from both proteasomal and lysosomal processing which resulted in accumulation of the protein inside and outside of the cells.

By measuring the polymerase activity in the lysates and cell culture fluids of the expressing cells, we found that introduction of the mutations in YMDD motif expected [42, 52] and confirmed here to completely abrogate the polymerase activity of the wild-type HIV-1 RT could not completely inactivate the multidrug-resistant enzyme. Both RT1.14opt-in and RT1.14oil preparations had a residual activity on the level of 0,0005% to 0,00005% of the activity of the equivalent amount of RT of HXB2 strain. Secretion of RT increased the biosafety, as we have observed that RT1.14oil was significantly less active than the parental RT1.14 both in the cells and when secreted in the cell culture medium. One possible explanation is in the RT-induced oxidative stress. Earlier, a phenomenon was described of the superoxide radical-mediated inactivation of an enzyme (myeloperoxidase) secreted by neutrophils [53]. Secreted RT1.14oil can undergo a likewise inactivation. Inactivation can also be due to the protein agglomeration following the accumulation of high amounts of undegradable RT1.14oil protein. We observed granular staining in the cytoplasm of RT1.14oil-expressing cells, and proteins of high molecular mass were specifically stained with anti-RT antibodies in Western blotting of cell lysates, the latter indicating formation of cross-linked protein agglomerates, similar to the observations made for the near-infrared reporter proteins [54]. The latter often results in the loss of enzymatic activity (protein function(s)) [55, 56]. Signal peptide-targeted secretion helped to diminish the residual polymerase activity and made RT1.14oil more attractive as the potential DNA vaccine component.

Artificial secretion resulted in a decrease of the capacity of RT to induce oxidative stress with 15% decrease in the production of ROS compared to the parental enzyme. This can be explained by the induction of the ARE-dependent mechanisms of oxidative stress response involving the enhanced expression of phase II detoxifying enzymes, specifically NAD(P)H-oxygenase 1. We have further inquired whether a decrease in the level of oxidative stress induced by RT1.14oil would improve its immunogenicity when delivered as DNA. We detected no significant enhancement of RT-specific immune response to RT1.14oil compared to RT1.14opt-in. Both genes induced a similar loss in the expression of luciferase reporter from the sites of immunization indicating that an immune response induced by RT-14opt-in and by RT1.14oil genes had a similar capacity for in vivo clearance of RT-expressing cells. The only difference was observed in the profile of the secreted effector molecules. Mice DNA immunized with RT1.14oil responded by the secretion of perforin, in which the latter correlated to the levels of RT-specific dual secretion of IFN-*γ*/IL-2. In contrast, mice DNA immunized with RT1.14opt-in responded by the RT-specific secretion of granzyme B not correlated to the production of either IFN-*γ* or IL-2. Thus, the enhancement of secretion due to signal peptide tag led to no major changes in the profile of anti-RT cellular immune response with the exception of a shift from the secretion of granzyme B in response to RT1.14opt-in, in favor of perforin in the response to RT1.14oil.

The addition of secretion signal is expected to promote antibody formation. Targeting for secretion of the membrane-bound glycoprotein of viral haematopoietic septicaemia virus resulted in the enhancement of IgM response in the rainbow trout [57]. DNA immunization with the cytoplasmic protein cathepsin B fused to the signal peptide of TPA induced a twofold increase in the level of specific IgG [58]. However, here, secretion of RT1.14 caused no enhancement of either IgG or IgA responses. Similar observations were made earlier for the rabies glycoprotein that was originally exposed on the cell surface and elicited strong antibody response even prior to artificial secretion; addition of a secretion signal gave no boost to the specific antibody response [59]. Apparently, even comparatively low amounts of extracellular immunogen (as in case of RT1.14opt-in) are capable of eliciting high antibody production. Further increase in the amounts of extracellular protein due to artificial secretion do not promote further enhancement of humoral responses. Of note, signal sequence of TBEV NS1 appeared to be nonimmunogenic on either cellular or antibody levels, the latter favoring its inclusion into the heterologous protein immunogens as a noninterfering transport moiety.

Both the parental and the signal peptide-tagged RTs were secreted. Enhancement of secretion due to signal peptide tag induced only moderate decrease in the oxidative stress. Hereby, we could extend our statement on the capacity of RTs to induce oxidative stress: it is induced by a wide panel of RT variants, wild-type and drug-resistant, expressed from viral and from the expression-optimized genes [19], preferably secreted as well as preferably intracellular, both enzymatically active and inactive. Altogether, this indicates that the propensity to induce oxidative stress and to modulate the immune response towards the antibody type is a property of the protein not linked to its enzymatic activities which can be modified and even abrogated.

We tentatively allocated this property to the RNase H domain of HIV-1 RT. RNases are necessary for the maintenance of cellular homeostasis, they participate in the degradation of ribosomes/cleavage of tRNA; RNases are involved in the recycling of phosphate during processes involving cell death and, hence, in the control of apoptosis [60]. Cleavage of tRNA by RNases is a conserved aspect of the response to oxidative stress [61, 62]. A secreted ribonuclease angiogenin selectively cleaves tRNA in eukaryotic cells; treatment of cells with this recombinant RNase or cell transfection with products of angiogenin activity (tRNA fragments) inhibits protein synthesis and induces apoptosis [62]. Overexpression of another RNase, RNase T2 family Rny1p, also caused apoptosis in yeast cells, whereas the deletion of its gene inhibited the apoptosis, specifically apoptosis in response to stress [63]. A proapoptotic activity of HIV-1 RNAse H was recently described [62]. Interestingly, Rny1p induced apoptosis independently of the catalytic activity through (yet unknown) interactions with the downstream components of the catabolic cell cascades [63, 64]. These observations indicate that oxidative stress induced by HIV RT expression may be linked to the properties of the RNase H moiety of the enzyme, not necessarily its activity. As Rny1p, inactive HIV-1 RNase H may retain the capacity to interact with the cellular proteins inducing oxidative stress and apoptotic cell death. By artificial secretion of HIV RT, we made it more stable and failed to reduce its content and, hence, the content of RNase H inside the cells, which in its turn did not allow us to significantly improve RT immunogenicity. Indirect confirmation of this concept lies in the experiments by Hallengard et al. who succeeded in raising strong immune response against RT after truncation of a part of RNase H domain [17]. Furthermore, we believe that the secretion that we found to be characteristic to this enzyme may play a role in the development of oxidative stress and oxidative stress response. Two other HIV proteins known to induce oxidative stress which contributes to HIV pathogenicity, namely, Tat and Vpr, are also secreted [65, 66]. Entry into the intercellular space turns Tat, Vpr, and possibly also RT into the signal molecules capable of affecting neighboring, also uninfected, cells. The actual mechanism(s) of the secretion of untagged RT and the input of this process into the induction of oxidative stress and modulation of immune response deserve a separate study.

## Supplementary Material

Supplementary Figure S1. Enzymatic activity of reverse transcriptase of HIV‐1 strain HXB2 (WT) and its variant inactivated by D185N, D186N and E478Q mutations (Mutant) purified as p51/p66 heterodimers in vitro tests of polymerase (A) and RNAse H activity (B, C). Influence of concentration of RT and inactivated RT on their RNase H activity visualized by the analysis of the products of RNAse H activity by PAGE (B); quantitative analysis of the activities (C). Solid dots and lines refer to the wild type, and hollow dots and dash lines, to inactivated RT. Figures represent the results of three independent experiments.

## Figures and Tables

**Table 1:** Expression, secretion, and residual enzymatic activity of inactivated forms of HIV reverse transcriptase RT1.14opt-in and RT1.14oil in transiently transfected HeLa cells. RT content was determined by Western blotting using protein calibration curves with signal quantification by ImageJ (dubbed WB, in *μ*g/10^5^ cells) and by quantification of enzymatic activity against standard RT preparation using Lenti RT activity test (Cavidi, Sweden) (dubbed EA, in pg/10^5^ cells). RT content in cell lysates is dubbed “in cells” and in cell culture fluids “secreted.” Specific activity was measured as a ratio of protein content evaluated using the enzymatic activity assay to RT content determined by Western blotting.

RT variant	RT content by WB, *μ*g/10^5^ cells			RT content by EA, pg/10^5^ cells			Specific RT activity (EA/WB), *μ*U	
	In cells	Secreted	Total	In cells	Secreted	Total	In cells	Secreted
RT1.14opt-in	8,30	14,00	22,30	9,80	74,40	85,00	1,18	5,30
RT1.14oil	11,30	34,60	46,00	5,70	4,90	11,00	0,51	0,14
