# Supplementary material for: Fusion to Flaviviral Leader Peptide Targets HIV-1 Reverse Transcriptase for Secretion and Reduces Its Enzymatic Activity and Ability to Induce Oxidative Stress but Has No Major Effects on Its Immunogenic Performance in DNA-Immunized Mice
Source: J Immunol Res. 2017 Jun 22;2017:7407136. doi: 10.1155/2017/7407136 (PMC5498913; doi:10.1155/2017/7407136)
Supplement: Supplementary file 1 — Supplementary Figure S1. Enzymatic activity of reverse transcriptase of HIV‐1 strain HXB2 (WT) and its variant inactivated by D185N, D186N and E478Q mutations (Mutant) purified as p51/p66 heterodimers in vitro tests of polymerase (A) and RNAse H activity (B, C). Influence of concentration of RT and inactivated RT on their RNase H activity visualized by the analysis of the products of RNAse H activity by PAGE (B); quantitative analysis of the activities (C). Solid dots and lines refer to the wild type, and hollow dots and dash lines, to inactivated RT. Figures represent the results of three independent experiments. [file 7407136.f1.pdf]

(A)

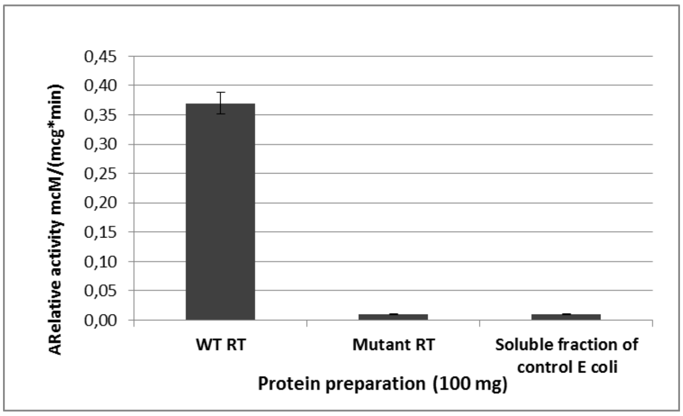

(B)

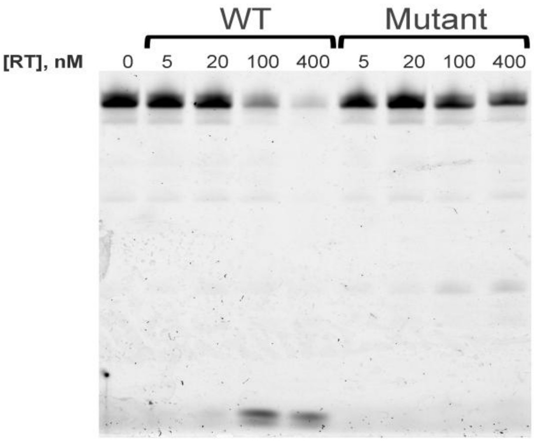

(C)

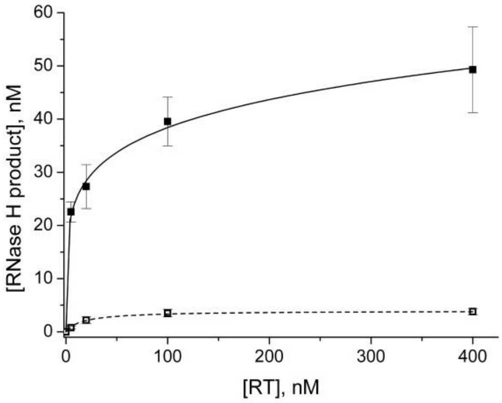

Supplementary Fig. S1 Enzymatic activity of reverse transcriptase of HIV-1 strain HXB2 (WT) and its variant inactivated by D185N, D186N and E478Q mutations (Mutant) purified as p51/p66 heterodimers *in vitro* tests of polymerase (A) and RNAse H activity (B, C). Influence of concentration of RT and inactivated RT on their RNase H activity visualized by the analysis of the products of RNAse H activity by PAGE (B); quantitative analysis of the activities (C). Solid dots and lines refer to the wild type, and hollow dots and dash lines, to inactivated RT. Figures represent the results of three independent experiments.
